# Epidemiological and performance indicators for occupational health services: a feasibility study in Belgium

**DOI:** 10.1186/1472-6963-14-410

**Published:** 2014-09-19

**Authors:** Lode Godderis, Kristien Johannik, Godewina Mylle, Simon Bulterys, Guido Moens

**Affiliations:** Groep IDEWE, External Service for Prevention and Protection at Work, Interleuvenlaan 58, Heverlee, 3001 Belgium; Centre for Environment and Health, Kapucijnenvoer 35/5, Leuven, 3000 Belgium

**Keywords:** Performance indicators, Epidemiology indicators, Occupational health services

## Abstract

**Background:**

In many European countries, Occupational Health and Safety (OHS) providers report their activities and results annually. Ideally, this report should offer an overview of their activities and of the outcome regarding occupational health and safety. To establish a set of epidemiological and performance indicators for electronic reporting of data that can be used for OHS surveillance and prevention purposes. Consequently, the selected data can serve as indicators for exposure to and prevention of occupational risks (epidemiology), and contribute to the evaluation of the functioning (performance) of OHS providers.

**Methods:**

An extensive literature search in combination with an investigation of existing reporting models was performed. The resulting list of potential indicators was assessed by different stakeholders and divided into indicators for epidemiology and for performance. Then in a feasibility study, the relevance and availability of the indicators were assessed in 17 external, 49 internal (in company) and 10 mixed OHS providers.

**Results:**

From the literature survey, we obtained 1100 indicators. After validation, 257 were taken into account in the feasibility study. An indicator was considered relevant when more than 2/3 of the respondents answered in favour of the indicator. The same criterion was applied for availability. Respectively, 82% and 62% of the performance and epidemiological indicators were considered to be relevant for external OHS providers. All relevant performance indicators were available. Of the epidemiological data, only 53% were available. Remarkably, internal OHS providers assessed fewer indicators as relevant (29% and 27% of performance and epidemiology indicators respectively), but these were mostly all available (90%).

**Conclusions:**

This study shows that it is possible to provide a snapshot of the state of OHS by means of the registration of data. These findings could be used to build a data warehouse to study national health and safety profiles and to develop a uniform report for all European countries.

**Electronic supplementary material:**

The online version of this article (doi:10.1186/1472-6963-14-410) contains supplementary material, which is available to authorized users.

## Background

In Europe, occupational health and safety is largely based on legislation, and more specifically on the implementation and adoption of the Framework Directive 89/391/EEC on Safety and Health at Work [1] which has an enormous impact on the tasks, methods and structures of Occupational Health and Safety (OHS) providers in European countries. OHS providers are services entrusted with essentially preventive functions. They are responsible for advising the employer, the workers and their representatives on the requirements for establishing and maintaining a safe and healthy work environment which will facilitate optimal physical and mental health in relation to work and the adaptation of work to the capabilities of workers [2]. Currently, Belgian OHS practice in general includes a broad list of preventive activities: workplace surveys, provision of information, counselling, health examinations, risk assessment, maintenance of first-aid skills, etc. The responsibilities, tasks and activities are for most European countries described in the national legislations [3–8].

In Belgium, all employers are obliged to provide occupational prevention services [9]. The Belgian law on Well-being at Work defines that occupational well-being in Belgium must be provided by ‘Services for Prevention and Protection at Work’ (SPPW). In this paper, we will use the term OHS provider, as this is more internationally accepted. Belgian OHS providers are by law divided into two units: (1) occupational health surveillance and (2) risk assessment and control [10]. Consequently, they must employ specialists from different fields: occupational physicians, ergonomists, safety specialists, psychosocial experts and hygienists.

An OHS provider can be set up by the employer and is hereafter called an ‘internal OHS provider’. In Belgium, about 10% of the companies, mostly large companies, have installed an internal SPPW and employ occupational physician, ergonomist, safety specialist, psychosocial expert and hygienist. Most employers, however, source out the prevention activities to so-called ‘external SPPW’ (hereafter called an ‘external OHS provider’). External OHS providers are independent of the employer and deliver services to 90% of the Belgian enterprises. In certain circumstances, employers can also choose to use an internal service while outsourcing some of the tasks to an external service (e.g. medical surveillance can be carried out by an internal service but certain risk management tasks can be outsourced to external services). These services are called ‘mixed SPPW’ and are hereafter called ‘mixed OHS providers’.

In many European countries, OHS providers report their activities and results annually. Ideally, this report should offer an overview of the activities of the organisation and of the outcome regarding occupational health and safety [11]. Hence, employers and employees organisations, health authorities, occupational health and safety professionals’ associations want to ascertain the quality, effectiveness and efficiency of OHS providers. In Belgium, each OHS provider has to submit an annual report of their activities to the Belgian authorities. Its content and format is defined by a royal decree. The authorities use this report to audit the performance of the OHS provider, in particular to assess whether an OHS provider has fulfilled its legal tasks. In addition, every Belgian OHS provider is audited by an independent certifying agency every three years following the ISO/DIS 9001:2007 criteria [12] in order to obtain the legal certification to provide occupational health services. Ideally, these audits should give an idea about the quantity and quality of the services rendered (performance). Unfortunately, the current focus is mainly on the quantity (number of examinations and assessments carried out) rather than on the quality (results of well-being policy or the extent to which providers meet the demands, needs and specifications of the customer) of the service delivered.

Belgian workers are divided into two groups depending on the presence or the absence of a well-defined occupational risk. Workers with occupational risks routinely undergo mandatory occupational health examinations, which include a pre-employment examination, a periodic medical examination and a work re-entrance examination after a four-week (or longer) sick leave. Each Belgian OHS provider has its own way of collecting data, mainly with the aim of compiling a medical file for personal monitoring and follow-up of an individual employee. This file contains administrative data, data about the worker's health (history, complaints, medication, sick leave, general health information, etc.) and work conditions (encoded risks, data about exposure, etc.). All these preventive actions and medical monitoring present a huge amount of data that could potentially be used to report trends on occupational health and work-related injuries. These data could be of interest for different purposes, such as working population OH surveillance, epidemiological studies and for personal monitoring of individual’s OH status.

In recent years, international interest in the registration of occupational health and safety data has increased [13, 14]. Moreover, occupational risks have changed over the last 50 years. The incidence of traditional occupational diseases such as pneumoconiosis, lead poisoning and hearing loss due to occupational exposure has decreased in developed countries. Simultaneously, however, the incidence of work-related musculoskeletal and psychological health problems has increased [15]. It is important that authorities use this information, but companies, sectors and OHS providers also need information on occupational health and safety. If both share the same needs and the selected items for reporting are relevant for both, the chance of obtaining accurate up-to-date information might increase significantly.

The main goal of this study was to establish a set of epidemiological and performance indicators for electronic reporting of data that can be used for OHS surveillance and prevention purposes using existing literature and expert review. “Epidemiological” OHS data could serve to create a national profile regarding well-being at work, which could be used in the development of a strategy on prevention and protection at work. “Performance” OHS data could be used for the operational evaluation and the cost-benefit analysis of the OHS provider [16–18]. We also investigated the feasibility of using the identified epidemiological and performance indicators by assessing the relevance and usefulness of these indicators to OHS providers. To our knowledge, this exercise has not been carried out in Belgium and other European countries and a set of OSH indicators needs to be established. These indicators should be parameters that deliver useful and usable data regarding exposure to and prevention of occupational risks (epidemiological indicators) and parameters for the operational evaluation of OHS (performance indicators). We hypothesise that the availability of data will be different depending on the type of OSH provider, e.g. internal or external.

## Methods

This project was commissioned by the Belgian Federal Government Service of Employment, Labour and Social affairs, and involved 3 steps to come to a comprehensive set of indicators: a literature review to select available indicators, an expert review to complement and assess the indicators, and finally a feasibility study in OHS providers to investigate the perceived relevancy and availability.

### Literature review

In order to produce a list of possible epidemiological and performance indicators measuring the primary goals of prevention and protection at work in OHS providers, an extensive literature search in Pubmed and an investigation of existing and previously developed national and international annual reports was performed through Google. We used following key terms: “occupational health, occupational medicine, definitions, indicators, work, prevention, safety”. Only papers published after the year 2000 were retained, and only indicators referring to the preventive tasks of occupational health services were included, thus excluding indicators referring to the treatment of occupational injuries or diseases.

We adopted the definition of an indicator as proposed in the European Community Health Indicators (ECHI) report [19]; namely, an indicator is “a concise definition of a concept, meant to provide maximal information on a specific area of interest”. We distinguished between epidemiological and performance indicators. Epidemiological indicators provide data on exposure to and prevention of occupational risks in the workplace and on health outcome while performance indicators are used to assess the operational functioning of the OHS.

As to epidemiological data, we did not opt for the traditional division between medical and non-medical activities or outcomes (diseases versus injuries). After a discussion with stakeholders and prevention counsellors with different expertise, a thematic classification was developed. The indicators were classified in such a way that each discipline could assign their relevant indicators to categories, including indicators of other fields.

The categories for epidemiological indicators were:

- Administrative data: gender, age, seniority, profession
- People and organisation: alcohol use, smoking habits, healthy lifestyle, bullying, physical workload, preventive medical examinations, etc.
- Chemical, physical, and biological agents
- Equipment and materials: working and technical certification, computer work, protective devices, manual handling of materials, etc.
- Working environment: fire and explosives, hygiene in healthcare, lightning, air, urgency planning, temperature, etc.
- Medication and sick leave
- Additional information

Performance indicators were further classified into four categories:

- Administrative data: number of enterprises, number of workplace health promotion specialists, etc.
- Input indicators evaluating the means, in terms of personnel and financial resources, of a specific program.
- Activity indicators quantifying the way in which services and goods are offered: information sessions given, benchmark comparisons, etc.
- Output indicators measuring the quantity of goods and services produced and the efficiency of the production (e.g. clients, activities): number of formal complaints, number of advices of prevention advisors, etc.

### Expert review

The initial list of 1100 indicators needed to be further completed in order to comply with the evolving legislation, with the specific demands of the Belgian authorities and also to reduce it to a number of practically implementable indicators. This was achieved by a needs analysis and revision of the list by several experts (academics in the field of OHS), users (Federal Public Service Employment, Labour and Social Dialogue and representatives of professional organisations in OHS) and stakeholders (representatives of social partners) in various meetings. In an iterative process the team of experts assessed the indicators on their validity, reliability, sensitivity, and specificity [2, 13]. In case of disagreement, experts presented and discussed the arguments until a final consensus was obtained. Validity implied that the indicator actually measured what it was supposed to measure. Reliability implied that even if the indicator was actually used by different people under the same circumstances, the results would be the same. Sensitivity meant that the indicator should be sensitive to changes in the situation or phenomenon concerned. Specificity meant that the indicator reflected changes only in the situation or phenomenon concerned.

### Feasibility study

To assess the relevance and availability of the 257 indicators identified after the literature and expert review, a feasibility study was set up in several OHS providers using the final list agreed on by the experts. An important attribute of an indicator is its availability, namely that it should be possible to obtain the required data without undue difficulty [2]. Relevance denotes how well the data meet the information needs of the user. The first user in this project is the authority, but companies and OHS providers also have a need for information on occupational health and safety. If both share the same needs and the selected indicators are relevant (yes/no) for both users, the chance of obtaining accurate up-to-date data might increase significantly.

We designed an electronic questionnaire. For each indicator on the final list (n = 257), we asked for its relevance (need to be mentioned in an annual report?) and its availability (can the indicator be measured?). This questionnaire was emailed to representatives of different types of OHS providers: internal, external or mixed services. The representatives were informed in advance during meetings and by means of an informative letter. The respective organisations involved were: COPREV (representing external OHS providers), PREBES (the board represents prevention advisors for companies and institutions; i.e. both internal and mixed OHS providers) and VVIB-AMTI (representing the occupational physicians of internal OHS providers). In total, 17 external, 49 internal and 10 mixed OHS providers were invited to complete the questionnaire. There was one respondent per OHS provider.

## Results

### Literature review

After revisions of the papers, we finally retained 12 publications containing OHS indicators [2, 13, 19–28], Based upon these international papers and reports, 1100 epidemiological and performance indicators were listed. Most of the indicators (39%) were useful for the evaluation of the performance of the OHS provider. Other data (34%) could produce potentially useful information for epidemiological goals. Further, indicators were classified as output (24%) or input (23%) indicators. Activity indicators formed the smallest group (12%). Some data (27%) were considered not relevant and were therefore not retained. The publication of Kreis and Bödeker [13] provided the largest number of indicators: 771 of which 38% were output and 30% were input indicators. Indicators were also divided according to their applicability in the context of external and/or internal OHS provider. 61% of the indicators were considered applicable for both external as well as internal OHS providers. A small set of indicators was only applicable for either external (4%) or internal/mixed services (8%).

### Expert review

After expert revision and completion with compliance indicators based on national (Codex of well-being at work) [9, 10] and international legislation, 257 indicators (25% of the original set of 1100 indicators) were retained for further analysis. Table 1 shows as an example a limited number of indicators with their reference and category. It also lists whether it is an epidemiological or performance indicator. The full list of 257 indicators for both epidemiological as well as performance indicators is available in Additional file 1). Because external and internal OHS providers are separate entities with a different organisation structure and objectives, different reporting modules were made for both services. Next, a further division was made according to the goals of the report: either the collection of epidemiological data or the collection of performance data. The categories covered in epidemiological and performance data were explained in the Methods section. For reporting medication and sick leave, the International Classification of Diseases (ICD)-10 code of the World Health Organization (WHO) was chosen.

**Table 1:** **Some examples of indicators and their reference**

Category	Indicator	Definition	Classification	External/internal OHS	Reference	Epidemiological/performance
**Administration**	NACE-Bel Code		Administration	I	Annual report enterprise	E
**Administration**	Financial	- % of total health expenditure,% of GNP/GDP	Administration	I	Kreis & Bödeker	P
**Administration**	Enterprises	- Total number of enterprises	Administration	E	Kreis & Bödeker	P
**Administration**	Occupational safety and health professionals	- Number of workplace health promotion specialists	Administration	E/I	OccHealth Indicators/Annual report/Good Practice	P
**Chemical, biological and physical agents**	Noise	- % of employees exposed to noise so loud that you would have to raise your voice to talk to people	Input	E/I	Kreis & Bödeker/PVI/OSHA	E
**Chemical, biological and physical agents**	Noise	- Noise induced hearing loss	Input	E/I	Kreis & Bödeker/PVI/OSHA	E
		- Tinnitus (permanent ringing in the ears)				
		- Threshold shift (initially temporary but becoming permanent with prolonged exposure)				
		- Loss of high frequency sounds resulting in communication problems				
		- Loss of interaction at social functions				
		- Noise exposure can also have secondary effects such as stress and interference with communication in the workplace causing injury events.				
**Individual and organisation**	Work rhythms	- Minutes per day normally spent travelling from home to work and back	Input	I	Kreis & Bödeker	E
**Individual and organisation**	Worked hours	- Days covered by employer	Input	E/I	Kreis & Bödeker	P
**Information sessions**	Information sessions given and followed	- Information sessions for employees	Activity	E/I	Kreis & Bödeker/Annual report/TWW/PVI	P
		- Information sessions for new employees				
**Information sessions**	Information sessions given and followed	- % of enterprises providing information on risks resulting from the working conditions	Activity	E/I	Kreis & Bödeker/Annual report/TWW/PVI	P
**Individual and organisation**	Financial	- Cost of lost working days due to sickness absence	Output	I	Kreis & Bödeker	P
**Individual and organisation**	Reintegration and disability management	- % of enterprises/institutions providing action on reintegration of staff (especially disabled staff) when they return to work after a longer-term period of sick-leave	Output	E/I	Kreis & Bödeker/Goodpractice/OccHealth Indicators	E
**Individual and organisation**	Reintegration and disability management	- % of partially disabled persons of working age in regular occupational activity (by cause, age, gender, occupation)	Output	E/I	Kreis & Bödeker/Goodpractice/OccHealth Indicators	E

### Feasibility study

The feasibility study was performed in 17 external (all), 49 internal (random selection) and 10 mixed (random selection) OHS providers. We obtained a fully completed questionnaire of respectively 9 (53%), 15 (31%), and 5 (50%) of these services. Indicators were considered to be relevant (yes/no) when more than 2/3 of the respondents answered positively. The same criterion was applied to assess the availability (yes/no). Table 2 gives an overview of the number of relevant indicators and their availability for each type of annual report. Additional file 1 contains the scored relevancy and availability of each indicator. We also found that relevance was not scored based on availability.

**Table 2:** **Overview of relevant indicators and their availability**

	Total	Number and percentage of relevant indicators	Number and percentage of non-relevant indicators	Number and percentage of relevant indicators available
**External services**	187	132 (71%)	55 (29%)	100 (76%)
**Epidemiologic**	109	68 (62%)	41 (38%)	36 (53%)
**Performance**	78	64 (82%)	14 (18%)	64 (100%)
**Internal services**	192	53 (28%)	139 (72%)	46 (87%)
**Epidemiologic**	108	29 (27%)	79 (73%)	26 (90%)
**Performance**	84	24 (29%)	60 (71%)	20 (83%)
**Mixed services**	99	19 (19%)	80 (81%)	13 (68%)

As to the evaluation of the performance of OHS providers, it is important to assess and interpret indicators taking into account other indicators. For this reason we created output indicators, which are mostly ratios of input and activity indicators. In Additional file 2, an overview of the performance indicators is given in an Excel file. The ratios are described and formulas programmed. The output indicators also make it possible to compare successive annual reports.

### Results for external OHS providers

External OHS providers found 68 (62%) of the 109 epidemiological indicators to be relevant (see Additional file 1). Of those, 36 (53%) were already available. The relevance of performance indicators was higher (64 out of 78 or 82%) and all of them were already available at that moment. In the context of the annual epidemiological report of external services, all indicators for the categories 'administration' and 'people and organisation' were relevant and available. More than 50% of the indicators from the categories 'chemical, physical, biological agents', 'fabrics and fibre', 'equipment and materials', 'working environment' and 'additional data', were noted as relevant. But only half of them were available. From the category 'agents', the results from workstations and the number of vaccinations for flu and varicella were not indicated as relevant. Similar results were found for the categories 'equipment and materials' and 'working environment', in which ergonomic results and results for measured workstations were not indicated as relevant. Indicators asking for new risks and the number of exposed employees were not found to be relevant. Although 50% of the indicators for medication and sick leave turned out to be relevant, these were not available for reporting. Indicators describing sick leave of employees were found to be relevant, medication use was not. Most of the indicators, proposed for the annual performance report of external services, were found to be relevant and were available as well. Within external services, turnover of clients and personnel turned out not to be relevant. Nevertheless, these figures were available.

### Results for internal OHS providers

Although most epidemiological indicators were similar for external (n = 187) and internal (n = 192) services, the response was remarkably different (see Additional file 1). For internal services, only 29 (27%) of the 108 epidemiological indicators turned out to be relevant. Of those, 26 (90%) were available at that moment. Also the relevance of performance indicators was relatively low (24 out of 84 or 29%). They were available for 83% (n = 20). For the epidemiological report of internal services, all indicators from the category 'people and organisation' were relevant and available. In other categories, relevance varied from 50% for administrative and additional information, over 30% for indicators regarding chemical, physical, biological agents, fabrics and fibre, equipment and materials to 0% for working environment and medication and sick leave. Availability of relevant indicators was high (80%-100%). In general, it can be stated that if indicators were not considered relevant for external services, they were also not relevant for internal services. The number of measured workstations from the categories 'agents', 'working environment' and 'equipment and materials' was not retained. Furthermore, internal occupational physicians found data regarding medication and sick leave not relevant. Those data were not available either.

Indicators evaluating performance differed significantly between external and internal services. For internal services, mainly the performance of the company (cf. mixed services) and to a lesser degree, the performance of the internal service, was measured. Therefore, questions regarding clients were left out for internal services. Questions regarding people and organisation (100%), additional information (100%) and prevention policy (78%) were mainly considered to be relevant and data were mostly available (>85%). There was less enthusiasm for the indicators in the other categories (relevance <34%). It was striking that questions regarding workstations and, more specifically, results from measurements, the outcome of the risk analysis and the already implemented measures, were not considered relevant nor were they available.

### Results for mixed OHS providers

Mixed OHS providers were only asked about performance indicators (see Additional file 1). Because of privacy aspects, epidemiological data of these services cannot be obtained. Only 19 out of 99 (19%) indicators turned out to be relevant, of which 13 (68%) were available at that time. Indicators used for the evaluation of performance differed significantly between external and mixed services, but corresponded with the performance indicators of internal services, mainly with the performance of the company. Further analysis per category revealed that the most relevant indicators could be found in the group of administration (40%), equipment and materials (40%) and people and organisation (50%). Availability of these relevant data turned out to be high (75-100%). For all other categories, relevance was lower than 34%. In general, indicators not relevant for external and internal OHS providers were not considered relevant by mixed services. Questions regarding workstations as well as results from measurements, the outcome of the risk analysis and implemented measures were not noted as relevant and data were not available either. Moreover, the benefit of reporting the present agents and the number of exposed employees was questioned. These data were also only partially available. No questions regarding new risks and procedures were retained.

## Discussion

Proposals of indicators have been made up according to the type of prevention service with common parts allowing the information to be combined. Despite the limitations, this study shows that it is possible to provide a comprehensive and factual snapshot of the state of occupational safety and health by means of the registration of data. If well designed, even a uniform report could be developed for all European countries. Annual reports can also present valuable information with respect to the sectors identified and discussed as being most at risk.

It is important that data are collected with one clear objective (epidemiological OR performance). If epidemiological data were used to evaluate the performance of the OHS provider, this could have consequences for the reliability of the data and could lead to underreporting. For this reason, the collection of epidemiological data was separated from the collection of performance data. Each set of data can be reported to the authorities and interested stakeholders.

As the feasibility study showed, not all of the indicators have been found to be relevant. Moreover, not all of the indicators were readily available. OHS providers often have their own registration system and there is currently no uniform way of registering data. For most of the risk factors, useful indicators could be found. However, it still remains difficult to measure and evaluate psychosocial factors. This project and proposal of indicators can be regarded as a first attempt to encourage authorities and OHS providers to develop a uniform system of registration.

Given the small numbers involved, we should be careful when drawing conclusions. It is, for instance, possible that results would be different if another respondent within the same OHS provider had participated. Thus, variability between observers within the same organisation could impair our findings. However, after analysing the data from different sources, results seemed to be congruous with each other. This finding suggests that these results largely represent the vision of the external, internal and mixed OHS providers.

It was somewhat difficult to make a clear-cut typology of indicators, whatever classification was used. We opted for a classification which could be used and understood by people with different OHS background and authorities. It is important to stress that each indicator is only meaningful within the total set of indicators. This means that the indicators should be interpreted as a whole. This is especially valid for performance indicators. All input indicators measure the means or the resources employed to facilitate the satisfaction of needs and, hence, reach development objectives. Output and outcome indicators measure the impact of a particular set of policies or a project on living standards of the population. Improvement in these types of indicators should determine the success of policies and projects as these try to measure the developmental impact. Output and outcomes should relate to objectives. The different levels of objectives justify the distinction between output and outcome.

Based on these limited number of observations, some findings are rather surprising. The relevance of most performance indicators was high and all of them were already available at that time. This is probably due to the fact that the management of the OHS provider used the same indicators to assess the OHS. Overall, the epidemiological indicators were judged not to be so relevant and remarkable differences were observed between the different types of OHS providers. In general, it seems that OHS providers do not see themselves as suppliers of epidemiological data, especially the internal OHS providers. Also more than 50% of the indicators from the categories 'chemical, physical, biological agents', 'fabrics and fibre', 'equipment and materials', 'working environment' and 'additional data', were noted as relevant. But only half of them were available. Especially surprising is the finding that indicators about new occupational risks were not considered as relevant, whereas this type of information is regarded as especially relevant by scientific, national and international organisations. Hence, the law stipulates that this as a task for OHS providers [9].

It is only meaningful to routinely register data about health and exposure, when a number of guidelines are followed for the optimal and qualitative registration and analysis of these data pertaining to health in the workplace [21, 22]. The goals of the registration as well as the choice, the standardisation of variables, quality and quality control (validity and reliability of the data), epidemiological methodology and applicability are all very important aspects of the registration. The question remains whether epidemiological data should be collected through the OHS providers. Hence, there might be huge observer variability, since the medical data are not registered for epidemiological purposes but rather for the individual follow-up of employees. Moreover, there may be little enthusiasm to register epidemiological data in a standardised manner. Therefore, another strategy for obtaining more valid and reliable data could be to work with sentinel occupational health physicians. Sentinel surveillance is the collection and analysis of data by motivated professionals or OHS providers selected according to their geographic location, occupational sector, medical specialty and ability to accurately diagnose and report high quality data. This procedure could also counter the lack of enthusiasm to record new occupational risks. Moreover, OHS providers do not exclusively decide what data should be registered; other stakeholders also have to play their role in the relevance and availability of indicators. The sentinel surveillance approach is currently used in the UK and Ireland and provides very valuable information, e.g. for the reporting of trends in occupational diseases [29–33]. Despite the advantages, it is not easy to set up and coordinate a sentinel system and the (voluntary) accurate collection of data also remains a challenge.

The registered items should be well standardised and reliable. A link should be established between objective exposure data, collected through the risk analysis, and subjective exposure data, obtained through medical examinations. ILO guidelines [22, 23] also mention a necessary link with environmental surveillance. The registration of objective exposure data can, in the long run, lead to a detailed inventory of the distribution of risks across various sectors and occupations. However, the study of the relationship between exposure and the prevalence of a certain health outcome is often complicated by a latency period between these two. As a consequence, there is a risk of selection bias (healthy worker effect) in the interpretation of detected signals. However, these signals collected through the system, could initiate more analytical and epidemiological studies conducted in a research setting. The system should also allow for primary preventive actions and interventions to be evaluated, again in a research environment. Finally, it is obvious that the data processing should be conducted according to the privacy legislation.

## Conclusion

We managed to establish a set of epidemiological and performance indicators for electronic reporting of data that can be used for OHS surveillance and prevention. For most of the risk factors, useful indicators could be found. As the feasibility study showed, not all of the indicators were assessed to be relevant by stakeholders. Moreover, not all of the indicators were readily available. OHS providers often have their own very specific registration system and there is no uniform way of registering data. This project and proposal of indicators can be regarded as a first attempt to encourage authorities and OHS providers to develop a uniform system of registration. Comparison of the reports during a certain period could allow for the monitoring of progress with a view to attaining a healthier and safer working environment.

## Electronic supplementary material

Additional file 1: **Full list of epidemiological and performance indicators for external, internal and mixed OHS providers.** (DOCX 101 KB)

Additional file 2: **Performance evaluation sheet for external OHS providers (including indicators, ratios and formulas).** (XLS 84 KB)
