# Supplementary material for: Epidemiological and performance indicators for occupational health services: a feasibility study in Belgium
Source: BMC Health Serv Res. 2014 Sep 19;14:410. doi: 10.1186/1472-6963-14-410 (PMC4180330; doi:10.1186/1472-6963-14-410)
Supplement: Supplementary file 1 — Additional file 1: Full list of epidemiological and performance indicators for external, internal and mixed OHS providers. (DOCX 101 KB) [file 12913_2013_3505_MOESM1_ESM.docx]

| EXTERNAL SERVICES | RELEVANT | AVAILABLE |
| --- | --- | --- |
| **EPIDEMIOLOGY** |  |  |
| **Administrative details** |  |  |
| Name of External Service | yes | yes |
| VAT number | yes | yes |
| Name of General Manager | yes | yes |
| **People and organisation** |  |  |
| Total number of employees (m/f) (subject to health surveillance (SHS) and not subject to health surveillance) | yes | yes |
| Number of employees (m/f) in shift work | yes | yes |
| Number of employees (m/f) with safety function | yes | yes |
| Number of employees (m/f) with increased vigilance | yes | yes |
| Number of informal complaints of undesirable conduct | yes | yes |
| Number of formal complaints of undesirable conduct | yes | yes |
| **Chemical, physical, biological agents, dust particles and fibres** |  |  |
| **Chemical agents** |  |  |
| Number of employees (m/f) exposed to chemical agents | yes | yes |
| Number of employees (m/f) exposed to carcinogens | yes | yes |
| Number of employees (m/f) exposed to mutagens | yes | no |
| Number of employees (m/f) exposed to teratogens | yes | no |
| Number of employees (m/f) exposed to dangerous substances | yes | no |
| Number of employees (m/f) exposed to irritants | no | no |
| Number of employees (m/f) exposed to toxic substances | yes | no |
| Number of employees (m/f) exposed to explosive substances | no | no |
| Number of employees (m/f) exposed to flammable substances | no | no |
| Number of biomonitoring investigations | yes | yes |
| Number of biomonitoring investigations with results above 50% of BEI | no | no |
| Number of biomonitoring investigations with results above BEI | yes | yes |
| Number of monitored work posts | yes | yes |
| Number of monitored work posts with exposure above 50% of Belgian threshold limit value | no | no |
| Number of monitored work posts with exposure above Belgian threshold limit value | yes | no |
| **Physical agents** |  |  |
| Number of employees (m/f) exposed to physical agents | yes | yes |
| Number of employees (m/f) exposed to noise levels greater than 80dB | yes | yes |
| Number of employees (m/f) exposed to vibrations | yes | yes |
| Number of employees (m/f) exposed to ionising radiation | yes | yes |
| Number of employees (m/f) exposed to non-ionising radiation | yes | yes |
| Number of employees (m/f) exposed to over/underpressure | yes | yes |
| Number of employees (m/f) exposed to electromagnetic fields | yes | yes |
| Number of monitored work posts exposed to noise | yes | yes |
| Number of monitored work posts exposed to noise levels greater than 80 dB | yes | no |
| Number of monitored work posts exposed to noise levels greater than 85 dB | yes | no |
| Number of monitored work posts exposed to noise levels greater than 87 dB | yes | no |
| Number of monitored work posts exposed to vibrations | yes | no |
| Number of monitored work posts exposed to vibrations above 50% of Belgian threshold limit value | no | no |
| Number of monitored work posts exposed to vibrations above Belgian threshold limit value | yes | no |
| Number of monitored work posts exposed to ionising radiation | yes | no |
| Number of monitored work posts exposed to non-ionising radiation | yes | no |
| Number of monitored work posts exposed to ionising radiation above 50% of Belgian threshold limit value | no | no |
| Number of monitored work posts exposed to non-ionising radiation above 50% of Belgian threshold limit value | no | no |
| Number of monitored work posts exposed to ionising radiation above Belgian threshold limit value | no | no |
| Number of monitored work posts exposed to non-ionising radiation above Belgian threshold limit value | no | no |
| Number of monitored work posts exposed to over/underpressure | no | no |
| Number of monitored work posts exposed to electromagnetic fields | no | no |
| **Biological agents** |  |  |
| Number of employees (m/f) vaccinated against hepatitis A | yes | yes |
| Number of employees (m/f) vaccinated against hepatitis B | yes | yes |
| Number of employees (m/f) vaccinated against chickenpox | no | no |
| Number of employees (m/f) vaccinated against influenza | no | yes |
| Number of employees (m/f) vaccinated against tetanus | yes | yes |
| **Dust particles and fibres** |  |  |
| Number of employees (m/f) exposed to particulate matter and nanoparticles | yes | no |
| Number of employees (m/f) exposed to asbestos fibres | yes | yes |
| Number of employees (m/f) exposed to man-made mineral fibres | yes | yes |
| Number of monitored work posts exposed to particulate matter and nanoparticles | yes | no |
| Number of monitored work posts exposed to particulate matter above 50% of Belgian threshold limit value | no | no |
| Number of monitored work posts exposed to particulate matter above Belgian threshold limit value | no | no |
| Number of monitored work posts with asbestos fibres | yes | yes |
| Number of monitored work posts exposed to asbestos fibres above 50% of Belgian threshold limit value | no | no |
| Number of monitored work posts exposed to asbestos fibres above the Belgian threshold limit value | yes | no |
| Number of monitored work posts with man-made mineral fibres | no | no |
| Number of monitored work posts exposed to man-made mineral fibres above 50% of Belgian threshold limit value | no | no |
| Number of monitored work posts exposed to man-made mineral fibres above the Belgian threshold limit value | no | no |
| **Equipment and materials** |  |  |
| **VDUs** |  |  |
| Number of employees (m/f) working with VDU | yes | yes |
| Number of ergonomically analysed VDU posts | yes | no |
| Number of ergonomically analysed VDU posts with health risks as a result of set-up | no | no |
| **Lifting loads** |  |  |
| Number of employees (m/f) exposed to lifting loads | yes | yes |
| Number of ergonomically analysed work posts | yes | no |
| Number of ergonomically analysed work posts with health risks as a result of set-up | no | no |
| **Work environment** |  |  |
| **Light and lighting** |  |  |
| Number of monitored work posts with light or lighting risks | yes | yes |
| Number of work posts with a luminance read-out below the threshold limit value | no | yes |
| **Temperature: cold/hot** |  |  |
| Number of monitored work posts in cold/hot conditions | yes | no |
| Number of monitored work posts with WBGT read-out above/under threshold limit value | no | no |
| **Additional information** |  |  |
| **Fitness for work** |  |  |
| Number of employees (m/f) with permanent unfitness for work | yes | yes |
| Number of employees (m/f) with temporary unfitness for work | yes | yes |
| Number of employees (m/f) with adapted work | yes | yes |
| Number of employees (m/f) sent for further examinations | yes | no |
| Number of employees (f) removed for maternity protection | yes | yes |
| **New risks** |  |  |
| Describe new procedures | yes | no |
| Describe new working conditions | no | no |
| Describe new dangers and risks | yes | no |
| Number of employees (m/f) exposed to new risks | no | no |
| **Medication and sick leave** |  |  |
| **Medication** |  |  |
| Number of employees (m/f) who are taking or have taken medication for a mental disorder | no | no |
| Number of employees (m/f) who are taking or have taken medication for neoplasms | no | no |
| Number of employees (m/f) who are taking or have taken medication for endocrine, metabolic or immunological disorders | no | no |
| Number of employees (m/f) who are taking or have taken medication for a disorder of the blood or blood-forming organs | no | no |
| Number of employees (m/f) who are taking or have taken medication for a complication during pregnancy or labour | no | no |
| Number of employees (m/f) who are taking or have taken medication for a disease of the skin or subcutaneous tissue | no | no |
| Number of employees (m/f) who are taking or have taken medication for a disease of the nervous system or sensory organs | no | no |
| Number of employees (m/f) who are taking or have taken medication for a disease of the blood or lymphatic system | no | no |
| Number of employees (m/f) who are taking or have taken medication for a musculoskeletal disorder or disorder of the connective tissue | no | no |
| Number of employees (m/f) who are taking or have taken medication for an infectious or parasitic disorder | no | no |
| Number of employees (m/f) who are taking or have taken medication for a disease of the digestive system | no | no |
| Number of employees (m/f) who are taking or have taken medication for a urogenital disease | no | no |
| Number of employees (m/f) who are taking or have taken medication for a respiratory disease | no | no |
| **Sick leave** |  |  |
| Number of employees (m/f) on sick leave as a result of a mental disorder | no | no |
| Number of employees (m/f) on sick leave as a result of a neoplasm | yes | no |
| Number of employees (m/f) on sick leave as a result of an endocrine, metabolic or immunological disorder | yes | no |
| Number of employees (m/f) on sick leave as a result of disorders of the blood or blood-making organs | yes | no |
| Number of employees (m/f) on sick leave as a result of a complication during pregnancy or labour | yes | no |
| Number of employees (m/f) on sick leave as a result of a disease of the skin or subcutaneous tissue | yes | no |
| Number of employees (m/f) on sick leave as a result of a disease of the nervous system or sensory organs | yes | no |
| Number of employees (m/f) on sick leave as a result of a disease of the blood or lymphatic system | yes | no |
| Number of employees (m/f) on sick leave as a result of a musculoskeletal disorder or disorder of the connective tissue | yes | no |
| Number of employees (m/f) on sick leave as a result of an infectious or parasitic disorder | yes | no |
| Number of employees (m/f) on sick leave as a result of a disease of the digestive system | yes | no |
| Number of employees (m/f) on sick leave as a result of a urogenital disease | yes | no |
| Number of employees (m/f) on sick leave as a result of a respiratory disease | yes | no |
| Number of employees (m/f) on sick leave as a result of a disorder that cannot be reported elsewhere | no | no |
| **PERFORMANCE** |  |  |
| **Administrative details** |  |  |
| Name of External Service | yes | yes |
| VAT number | yes | yes |
| Name of General Manager | yes | yes |
| Address of registered headquarters | yes | yes |
| Phone number of registered headquarters | yes | yes |
| Website URL | no | yes |
| **Personnel in External Service** |  |  |
| Total number of employees (m/f) | yes | yes |
| Total number of new employees (m/f) | no | yes |
| Total number of employees (m/f) on notice | no | yes |
| **Certified Health & Safety Officers** |  |  |
| Total number of certified H&S officers for Occupational Health | yes | yes |
| Total number of certified H&S officers for Ionising radiation | yes | yes |
| Total number of certified H&S officers for Occupational Hygiene | yes | yes |
| Total number of certified H&S officers for Occupational Safety | yes | yes |
| Total number of certified H&S officers for Ergonomics | yes | yes |
| Total number of certified H&S officers for Psychosocial factors | yes | yes |
| **H&S officers in training** |  |  |
| Total number of certified H&S officers in training for Occupational Health | yes | yes |
| Total number of certified H&S officers in training for Ionising radiation | yes | yes |
| Total number of certified H&S officers in training for Occupational Hygiene | yes | yes |
| Total number of certified H&S officers in training for Occupational Safety | yes | yes |
| Total number of certified H&S officers in training for Ergonomics | yes | yes |
| Total number of certified H&S officers in training for Psychosocial factors | yes | yes |
| **H&S employees** |  |  |
| Total number of nurses | yes | yes |
| Total number of nurses with additional training | yes | yes |
| Total number of assistant H&S officers | yes | yes |
| Total number of assistant H&S officers with additional training | yes | yes |
| **Additional courses followed (per half day)** |  |  |
| Occupational hygiene | yes | yes |
| Ergonomics | yes | yes |
| Health | yes | yes |
| Psychosocial | yes | yes |
| Safety | yes | yes |
| **Clients External Service** |  |  |
| **Companies** |  |  |
| Affiliated companies (type A, B, C, D) | yes | yes |
| Recently affiliated companies (type A, B, C, D) | yes | yes |
| Companies on notice (type A, B, C, D) | no | yes |
| Passive companies (type A, B, C, D) | no | no |
| **Employees** |  |  |
| Number of (annually) SHS employees (m/f) (type A, B, C, D) | yes | yes |
| Number of employees (m/f) ionising radiation (type A, B, C, D) | no | yes |
| Number of (3-yearly) SHS employees (m/f) (type A, B, C, D) | no | yes |
| Number of (5-yearly) SHS employees (m/f) (type A, B, C, D) | no | yes |
| Number of non-SHS employees (m/f) (type A, B, C, D) | yes | yes |
| **Activities of External Service** |  |  |
| **Committees** |  |  |
| Number of committees attended for Health & Safety at work (type A, B, C) | yes | yes |
| **Health assessment (SHS, non-SHS)** |  |  |
| Number of prior health assessments | yes | yes |
| Number of periodic health assessments | yes | yes |
| Number of health assessments for resumption of work | yes | yes |
| Number of health assessments after resumption of work | yes | yes |
| Number of spontaneous health assessments | yes | yes |
| Number of health assessments for re-integration | yes | yes |
| Number of health assessments for maternity protection | yes | yes |
| Number of health assessments for application to fund for occupational illnesses | yes | yes |
| Number of health assessments for application for occupational injury event | yes | yes |
| Number of health assessments at request of employer | yes | yes |
| Number of health assessments carried out | yes | yes |
| **Other health assessments** |  |  |
| Driving proficiency certificate | yes | yes |
| Check-ups | no | no |
| **Company visits/risk analyses (A,B,C,D companies) (per half day)** |  |  |
| BB/RA Occupational hygiene (A,B,C,D companies) | yes | yes |
| BB/RA Ergonomics (A,B,C,D companies) | yes | yes |
| BB/RA Health (A,B,C,D companies) | yes | yes |
| BB/RA Psychosocial (A,B,C,D companies) | yes | yes |
| BB/RA Safety (A,B,C,D companies) | yes | yes |
| **Performance** |  |  |
| Number of companies that have received no report for mandatory company visit in the last 3 years | no | yes |
| Number of SHS employees who have not undergone a health assessment in the last 2 years | no | yes |
| **Registered environmental readings** |  |  |
| Environmental readings for physical agents | yes | yes |
| Environmental readings for chemical agents | yes | yes |
| Environmental readings for biological agents | no | no |
| Environmental readings for dust particles and fibres | yes | yes |
| **Courses (number of half work days)** |  |  |
| Occupational hygiene course | yes | yes |
| Ergonomics course | yes | yes |
| Health course | yes | yes |
| Psychosocial course | yes | yes |
| Safety course | yes | yes |
| **Information sessions (per half day)** |  |  |
| Occupational hygiene information session | yes | yes |
| Ergonomics information session | yes | yes |
| Health information session | yes | yes |
| Psychosocial information session | yes | yes |
| Safety information session | yes | yes |
| **Scientific activities** |  |  |
| Non-peer-reviewed publications | no | yes |
| Peer-reviewed publications | no | no |
| Participation in seminars and courses (per half day) | yes | yes |
| Talk or poster contribution at seminar | yes | yes |

| INTERNAL SERVICES | RELEVANT | AVAILABLE |
| --- | --- | --- |
| **Administrative details** |  |  |
| VAT number | no | yes |
| NACE(BEL)code | no | yes |
| Type of company | yes | yes |
| Name of director | no | yes |
| Total number of employees (m/f) with permanent contract | no | yes |
| Total number of employees (m/f) with temporary contract | no | yes |
| Total number of employees (m/f) with interim contract | no | yes |
| Total number of external employees (m/f) (third parties) | no | no |
| Total number of employees (m/f) who have left the company this year | no | yes |
| Total number of employees (m/f) recruited this year | no | yes |
| **Health & Safety policy** |  |  |
| Number of committees for workplace Health & Safety | yes | yes |
| Education level of H&S officer | yes | yes |
| Name, specialism and employment grade of internal certified H&S officers | yes | yes |
| External Service for Workplace Health and Safety | yes | yes |
| Name, specialism and employment grade of external certified H&S officers | no | yes |
| List certificates, quality labels, care systems, ... (OHSAS, ISO, ...) | no | no |
| Number of visits made to work place (with report) | yes | yes |
| Number of risk analyses carried out (with report); Subject reported: safety, health, ergonomics, hygiene, psychosocial | yes | yes |
| **Courses and training** |  |  |
| VCA (*safety check for contractors*), first aid, emergency plan and evacuation, stop smoking, alcohol and drugs, management of unacceptable conduct, HACCP, new employees, hierarchy, lifting loads | yes | no |
| **People and organisation** |  |  |
| Is there a policy on psychosocial factors? | yes | yes |
| Number of employees (m/f) with adapted work as a result of work injury event or medical impairment | yes | yes |
| **Chemical, physical, biological agents** |  |  |
| Agents present: chemical agents, physical agents, biological agents, dust particles and fibres | yes | yes |
| **Chemical agents** |  |  |
| Chemical risks present: carcinogens, mutagens, teratogens, dangerous substances, irritants, toxic substances, explosive substances, flammable substances, environmental pollutants, particulate matter and nanoparticles | yes | yes |
| Lowest prevention management level | no | no |
| % work posts in reported worst-case conditions | no | no |
| % work posts where personal protective equipment must be worn | no | no |
| Number of employees that must wear personal protective equipment | no | no |
| **Physical agents** |  |  |
| Physical risks present: noise, vibrations, radiation, electromagnetic fields | yes | yes |
| **Physical agents: Noise** |  |  |
| Highest exposure level in dB | no | yes |
| Lowest prevention management level | no | no |
| % work posts in reported worst-case conditions | no | no |
| **Physical agents: vibrations** |  |  |
| Vibrations present: global body vibrations, hand-arm vibrations | yes | yes |
| Lowest prevention management level | no | no |
| % work posts in reported worst-case conditions | no | no |
| **Physical agents: radiation** |  |  |
| Radiation present: ionising radiation, non-ionising radiation | yes | yes |
| Lowest prevention management level | no | no |
| Number of employees (m/f) exposed to radiation | yes | yes |
| % work posts where personal protective equipment must be worn | no | no |
| Number of employees that must wear personal protective equipment | no | no |
| **Biological agents** |  |  |
| Biological risks present: group 1, group 2, group 3, group 4 | yes | yes |
| Lowest prevention management level | no | no |
| % work posts in reported worst-case conditions | no | no |
| Number of employees (m/f) exposed to biological agents | yes | yes |
| % work posts where personal protective equipment must be worn | no | no |
| Number of employees that must wear personal protective equipment | no | no |
| **Dust particles and fibres** |  |  |
| Dust particles and fibres present: total dust particles, nanoparticles, asbestos fibres, man-made mineral fibres | yes | yes |
| Lowest prevention management level | no | no |
| % work posts in reported worst-case conditions | no | no |
| Number of employees (m/f) exposed to dust particles and fibres | no | yes |
| % work posts where personal protective equipment must be worn | no | no |
| Number of employees that must wear personal protective equipment | no | no |
| **Equipment and materials** |  |  |
| Present circumstances: VDUs, lifting loads, work posts and tasks where personal protective equipment must be worn | yes | no |
| **Lifting loads** |  |  |
| Lowest prevention management level | no | no |
| % work posts in reported worst-case conditions | no | no |
| **Work environment** |  |  |
| Dangers present: fire and/or explosion, light and lighting, temporary mobile construction sites, temperature, height, moving parts, electricity, enclosed spaces, other, none | yes | no |
| **Fire and/or explosion** |  |  |
| Number of work posts with fire and/or explosion risk | no | no |
| Lowest prevention management level | no | no |
| % work posts in reported worst-case conditions | no | no |
| **Light and lighting** |  |  |
| Lowest prevention management level | no | no |
| % work posts in reported worst-case conditions | no | no |
| **Temporary mobile construction sites** |  |  |
| Number of temporary mobile construction sites | no | no |
| Lowest prevention management level | no | no |
| % work posts in reported worst-case conditions | no | no |
| **Temperature: cold/hot** |  |  |
| Number of work posts in cold/hot conditions | yes | yes |
| Lowest prevention management level | no | no |
| % work posts in reported worst-case conditions | no | no |
| **Height** |  |  |
| Number of work posts at great height | no | no |
| Lowest prevention management level | no | no |
| % work posts in reported worst-case conditions | no | no |
| **Moving parts** |  |  |
| Number of work posts with moving parts | no | no |
| Lowest prevention management level | no | no |
| % work posts in reported worst-case conditions | no | no |
| **Electricity** |  |  |
| Number of work posts with electrical risks | yes | no |
| Lowest prevention management level | no | no |
| % work posts in reported worst-case conditions | no | no |
| number of BA4 electricians | no | no |
| number of BA5 electricians | no | no |
| **Enclosed spaces** |  |  |
| Number of work posts with enclosed spaces | no | no |
| Lowest prevention management level | no | no |
| % work posts in reported worst-case conditions | no | no |
| **Other** |  |  |
| Other risks | no | no |
| Lowest prevention management level | no | no |
| % work posts in reported worst-case conditions | no | no |
| **Additional information: Describe below procedures, risks, working conditions that are not mentioned above** |  |  |
| Describe additional activities carried out in the context of health and safety at work | yes | yes |
| **EPIDEMIOLOGY** |  |  |
| **Administrative details** |  |  |
| Name of Internal Service | yes | yes |
| Name of General Manager | no | yes |
| **People and organisation** |  |  |
| Total number of employees (m/f) (subject to health surveillance (SHS) and not subject to health surveillance) | yes | yes |
| Number of employees (m/f) in shift work | yes | yes |
| Number of employees (m/f) with safety function | yes | yes |
| Number of employees (m/f) with increased vigilance | yes | yes |
| Number of informal complaints about undesirable conduct | yes | yes |
| Number of complaints about undesirable conduct | yes | yes |
| **Chemical, physical, biological agents, dust particles and fibres** |  |  |
| **Chemical agents** |  |  |
| Number of employees (m/f) exposed to chemical agents | yes | yes |
| Number of employees (m/f) exposed to carcinogens | yes | yes |
| Number of employees (m/f) exposed to mutagens | yes | yes |
| Number of employees (m/f) exposed to teratogens | yes | no |
| Number of employees (m/f) exposed to dangerous substances | no | yes |
| Number of employees (m/f) exposed to irritants | no | no |
| Number of employees (m/f) exposed to toxic substances | no | yes |
| Number of employees (m/f) exposed to explosive substances | no | no |
| Number of employees (m/f) exposed to flammable substances | no | no |
| Number of biomonitoring investigations | yes | yes |
| Number of biomonitoring investigations with results above 50% of BEI | no | no |
| Number of biomonitoring investigations with results above BEI | no | no |
| Number of monitored work posts | no | no |
| Number of monitored work posts with exposure above 50% of Belgian threshold limit value | no | no |
| Number of monitored work posts with exposure above Belgian threshold limit value | no | no |
| **Physical agents** |  |  |
| Number of employees (m/f) exposed to physical agents | yes | yes |
| Number of employees (m/f) exposed to noise levels greater than 80dB | yes | yes |
| Number of employees (m/f) exposed to vibrations | yes | yes |
| Number of employees (m/f) exposed to ionising radiation | yes | yes |
| Number of employees (m/f) exposed to non-ionising radiation | no | yes |
| Number of employees (m/f) exposed to over/underpressure | yes | yes |
| Number of employees (m/f) exposed to electromagnetic fields | no | no |
| Number of monitored work posts exposed to noise | no | yes |
| Number of monitored work posts exposed to noise levels greater than 80 dB | no | yes |
| Number of monitored work posts exposed to noise levels greater than 85 dB | no | yes |
| Number of monitored work posts exposed to noise levels greater than 87 dB | no | no |
| Number of monitored work posts exposed to vibrations | no | no |
| Number of monitored work posts exposed to vibrations above 50% of Belgian threshold limit value | no | no |
| Number of monitored work posts exposed to vibrations above Belgian threshold limit value | no | no |
| Number of monitored work posts exposed to ionising radiation | no | yes |
| Number of monitored work posts exposed to non-ionising radiation | no | no |
| Number of monitored work posts exposed to ionising radiation above 50% of Belgian threshold limit value | no | no |
| Number of monitored work posts exposed to non-ionising radiation above 50% of Belgian threshold limit value | no | no |
| Number of monitored work posts exposed to ionising radiation above Belgian threshold limit value | no | no |
| Number of monitored work posts exposed to non-ionising radiation above Belgian threshold limit value | no | no |
| Number of monitored work posts exposed to over/underpressure | no | no |
| Number of monitored work posts exposed to electromagnetic fields | no | no |
| **Biological agents** |  |  |
| Number of employees (m/f) vaccinated against hepatitis A | yes | yes |
| Number of employees (m/f) vaccinated against hepatitis B | yes | yes |
| Number of employees (m/f) vaccinated against chickenpox | no | no |
| Number of employees (m/f) vaccinated against influenza | no | yes |
| Number of employees (m/f) vaccinated against tetanus | yes | yes |
| **Dust particles and fibres** |  |  |
| Number of employees (m/f) exposed to particulate matter and nanoparticles | no | no |
| Number of employees (m/f) exposed to asbestos fibres | yes | yes |
| Number of employees (m/f) exposed to man-made mineral fibres | yes | no |
| Number of monitored work posts exposed to particulate matter and nanoparticles | no | no |
| Number of monitored work posts exposed to particulate matter above 50% of Belgian threshold limit value | no | no |
| Number of monitored work posts exposed to particulate matter above Belgian threshold limit value | no | no |
| Number of monitored work posts with asbestos fibres | no | no |
| Number of monitored work posts exposed to asbestos fibres above 50% of Belgian threshold limit value | no | no |
| Number of monitored work posts exposed to asbestos fibres above the Belgian threshold limit value | no | no |
| Number of monitored work posts with man-made mineral fibres | no | no |
| Number of monitored work posts exposed to man-made mineral fibres above 50% of Belgian threshold limit value | no | no |
| Number of monitored work posts exposed to man-made mineral fibres above the Belgian threshold limit value | no | no |
| **Equipment and materials** |  |  |
| **VDUs** |  |  |
| Number of employees (m/f) working with VDU | yes | yes |
| Number of ergonomically analysed VDU posts | no | no |
| Number of ergonomically analysed VDU posts with health risks as a result of set-up | no | no |
| **Lifting loads** |  |  |
| Number of employees (m/f) exposed to lifting loads | yes | yes |
| Number of ergonomically analysed work posts | no | no |
| Number of ergonomically analysed work posts with health risks as a result of set-up | no | no |
| **Work environment** |  |  |
| **Light and lighting** |  |  |
| Number of monitored work posts with light or lighting risks | no | no |
| Number of work posts with a luminance read-out below the threshold limit value | no | no |
| **Temperature: cold/hot** |  |  |
| Number of monitored work posts in cold/hot conditions | no | no |
| Number of monitored work posts with monitored WBGT above/under threshold limit value | no | no |
| **Additional information** |  |  |
| **Fitness for work** |  |  |
| Number of employees (m/f) with permanent unfitness for work | yes | yes |
| Number of employees (m/f) with temporary unfitness for work | yes | yes |
| Number of employees (m/f) with adapted work | yes | yes |
| Number of employees (m/f) sent for further examinations | yes | no |
| Number of employees (f) removed for maternity protection | yes | yes |
| **New risks** |  |  |
| Describe new procedures | no | no |
| Describe new working conditions | no | no |
| Describe new dangers and risks | no | no |
| Number of employees (m/f) exposed to new risks | no | no |
| **Medication and sick leave** |  |  |
| **Medication** |  |  |
| Number of employees (m/f) who are taking or have taken medication for a mental disorder | no | no |
| Number of employees (m/f) who are taking or have taken medication for neoplasms | no | no |
| Number of employees (m/f) who are taking or have taken medication for endocrine, metabolic or immunological disorders | no | no |
| Number of employees (m/f) who are taking or have taken medication for a disorder of the blood or blood-forming organs | no | no |
| Number of employees (m/f) who are taking or have taken medication for a complication during pregnancy or labour | no | no |
| Number of employees (m/f) who are taking or have taken medication for a disease of the skin or subcutaneous fibre | no | no |
| Number of employees (m/f) who are taking or have taken medication for a disease of the nervous system or sensory organs | no | no |
| Number of employees (m/f) who are taking or have taken medication for a disease of the blood or lymphatic system | no | no |
| Number of employees (m/f) who are taking or have taken medication for a musculoskeletal disorder or disorder of the connective tissue | no | no |
| Number of employees (m/f) who are taking or have taken medication for an infectious or parasitic disorder | no | no |
| Number of employees (m/f) who are taking or have taken medication for a disease of the digestive system | no | no |
| Number of employees (m/f) who are taking or have taken medication for a urogenital disease | no | no |
| Number of employees (m/f) who are taking or have taken medication for a respiratory disease | no | no |
| **Sick leave** |  |  |
| Number of employees (m/f) on sick leave as a result of a mental disorder | no | no |
| Number of employees (m/f) on sick leave as a result of a neoplasm | no | no |
| Number of employees (m/f) on sick leave as a result of an endocrine, metabolic or immunological disorder | no | no |
| Number of employees (m/f) on sick leave as a result of disorders of the blood or blood-making organs | no | no |
| Number of employees (m/f) on sick leave as a result of a complication during pregnancy or labour | no | no |
| Number of employees (m/f) on sick leave as a result of a disease of the skin or subcutaneous tissue | no | no |
| Number of employees (m/f) on sick leave as a result of a disease of the nervous system or sensory organs | no | no |
| Number of employees (m/f) on sick leave as a result of a disease of the blood or lymphatic system | no | no |
| Number of employees (m/f) on sick leave as a result of a musculoskeletal disorder or disorder of the connective tissue | no | no |
| Number of employees (m/f) on sick leave as a result of an infectious or parasitic disorder | no | no |
| Number of employees (m/f) on sick leave as a result of a disease of the digestive system | no | no |
| Number of employees (m/f) on sick leave as a result of a urogenital disease | no | no |
| Number of employees (m/f) on sick leave as a result of a respiratory disease | no | no |
| Number of employees (m/f) on sick leave as a result of symptoms or a disorder that cannot be reported elsewhere | no | no |

| MIXED SERVICES | RELEVANT | AVAILABLE |
| --- | --- | --- |
| **Administrative details** |  |  |
| VAT number. | no | no |
| NACE(BEL)code. | yes | yes |
| Type of company. | yes | no |
| Name of director. | yes | yes |
| Total number of employees (m/f) with permanent contract. | yes | yes |
| Total number of employees (m/f) with temporary contract. | no | yes |
| Total number of employees (m/f) with interim contract. | no | no |
| Total number of external employees (m/f) (third parties). | no | no |
| Total number of employees (m/f) who have left the company this year. | no | yes |
| Total number of employees (m/f) recruited this year. | no | yes |
| **Health and Safety policy** |  |  |
| Number of committees for workplace health and safety. | yes | yes |
| Education level of H&S officer. | yes | yes |
| Name, specialism and employment grade of internal certified H&S officers. | no | yes |
| External Service for Workplace Health and Safety | no | no |
| Name, specialism and employment grade of external certified H&S officers | no | no |
| List certificates, quality labels, care systems, ... (OHSAS, ISO, ...) | no | yes |
| Number of visits made to work place (with report) | no | yes |
| Number of risk analyses carried out (with report); Subject reported: safety, health, ergonomics, hygiene, psychosocial | no | yes |
| **Courses and training** |  |  |
| VCA (*safety check for contractors*), first aid, emergency plan and evacuation, stop smoking, alcohol and drugs, management of unacceptable conduct, HACCP, new employees, hierarchy, lifting loads | yes | yes |
| **People and organisation** |  |  |
| Number of employees (m/f) in shift work | no | yes |
| Number of employees (m/f) with safety function | no | yes |
| Number of informal complaints about undesirable conduct | no | no |
| Number of formal complaints about undesirable conduct | yes | yes |
| Is there a policy on psychosocial factors? | yes | yes |
| Number of employees (m/f) with permanent unfitness for work | yes | yes |
| Number of employees (m/f) with temporary unfitness for work | no | yes |
| Number of employees (m/f) with adapted work as a result of work injury event or medical impairment | yes | yes |
| **Chemical, physical, biological agents** |  |  |
| Agents present: chemical agents, physical agents, biological agents, dust particles and fibres, none | no | yes |
| **Chemical agents** |  |  |
| Chemical risks present: carcinogens, mutagens, teratogens, dangerous substances, irritants, toxic substances, explosive substances, flammable substances, environmental pollutants, particulate matter and nanoparticles | yes | yes |
| Lowest prevention management level | no | no |
| % work posts in reported worst-case conditions | no | no |
| Number of employees (m/f) exposed to chemical agents | no | yes |
| % work posts where personal protective equipment must be worn | yes | no |
| Number of employees that must wear personal protective equipment | no | no |
| **Physical agents** |  |  |
| Physical risks present: noise, vibrations, radiation, electromagnetic fields | yes | yes |
| **Physical agents: Noise** |  |  |
| Highest exposure level in dB | no | no |
| Lowest prevention management level | no | no |
| % work posts in reported worst-case conditions | no | no |
| Number of employees (m/f) exposed to noise levels greater than 80dB | no | no |
| **Physical agents: vibrations** | no | no |
| Vibrations present: global body vibrations, hand-arm vibrations | no | no |
| Lowest prevention management level | no | no |
| % work posts in reported worst-case conditions | no | no |
| Number of employees (m/f) exposed to vibrations | no | no |
| **Physical agents: radiation** |  |  |
| Radiation present: ionising radiation, non-ionising radiation | yes | yes |
| Lowest prevention management level | no | no |
| Number of employees (m/f) exposed to radiation | no | no |
| % work posts where personal protective equipment must be worn | no | no |
| Number of employees that must wear personal protective equipment | no | no |
| **Biological agents** |  |  |
| Biological risks present: group 1, group 2, group 3, group 4. | yes | no |
| Lowest prevention management level | no | no |
| % work posts in reported worst-case conditions | no | no |
| Number of employees (m/f) exposed to biological agents | no | no |
| % work posts where personal protective equipment must be worn | no | no |
| Number of employees that must wear personal protective equipment | no | no |
| **Dust particles and fibres** |  |  |
| Dust particles and fibres present: total dust particles, nanoparticles, asbestos fibres, man-made mineral fibres | no | no |
| Lowest prevention management level | no | no |
| % work posts in reported worst-case conditions | no | no |
| Number of employees (m/f) exposed to dust particles and fibres | no | no |
| % work posts where personal protective equipment must be worn | no | no |
| Number of employees that must wear personal protective equipment | no | no |
| **Equipment and materials** |  |  |
| Present circumstances: VDUs, lifting loads, work posts and tasks where personal protective equipment must be worn | yes | no |
| **VDUs** |  |  |
| Number of employees (m/f) working with VDU | yes | no |
| **Lifting loads** |  |  |
| Lowest prevention management level | no | no |
| % work posts in reported worst-case conditions | no | no |
| Number of employees (m/f) exposed to lifting loads | no | no |
| **Work environment** |  |  |
| Dangers present: fire and/or explosion, light and lighting, temporary mobile construction sites, temperature, height, moving parts, electricity, enclosed spaces, other, none | yes | no |
| **Fire and/or explosion** |  |  |
| Number of work posts with fire and/or explosion risk | no | no |
| Lowest prevention management level | no | no |
| % work posts in reported worst-case conditions | no | no |
| **Light and lighting** |  |  |
| Number of work posts with light or lighting risks | no | no |
| Lowest prevention management level | no | no |
| % work posts in reported worst-case conditions | no | no |
| **Temporary mobile construction sites** |  |  |
| Number of temporary mobile construction sites | no | no |
| Lowest prevention management level | no | no |
| % work posts in reported worst-case conditions | no | no |
| **Temperature: cold/hot** |  |  |
| Number of work posts in cold/hot conditions | no | no |
| Lowest prevention management level | no | no |
| % work posts in reported worst-case conditions | no | no |
| **Height** |  |  |
| Number of work posts at great height | no | no |
| Lowest prevention management level | no | no |
| % work posts in reported worst-case conditions | no | no |
| **Moving parts** |  |  |
| Number of work posts with moving parts | no | no |
| Lowest prevention management level | no | no |
| % work posts in reported worst-case conditions | no | no |
| **Electricity** |  |  |
| Number of work posts with electrical risks | no | no |
| Lowest prevention management level | no | no |
| % work posts in reported worst-case conditions | no | no |
| number of BA4 electricians | no | no |
| number of BA5 electricians | no | no |
| **Enclosed spaces** |  |  |
| Number of work posts with enclosed spaces | no | no |
| Lowest prevention management level | no | no |
| % work posts in reported worst-case conditions | no | no |
| **Other** |  |  |
| Other risks | no | no |
| Lowest prevention management level | no | no |
| % work posts in reported worst-case conditions | no | no |
| **Additional information** |  |  |
| Describe new procedures | no | no |
| Describe new working conditions | no | no |
| Describe new dangers and risks | no | no |
| Describe additional activities carried out in the context of workplace health and safety | no | no |
